# Roles of Crosstalk between Astrocytes and Microglia in Triggering Neuroinflammation and Brain Edema Formation in 1,2-Dichloroethane-Intoxicated Mice

**DOI:** 10.3390/cells10102647

**Published:** 2021-10-03

**Authors:** Jinhan Yang, Tong Wang, Xiaoxia Jin, Gaoyang Wang, Fenghong Zhao, Yaping Jin

**Affiliations:** 1 Department of Environmental and Occupational Health, School of Public Health, China Medical University, Shenyang 110122, China; jhyang2211@outlook.com (J.Y.); wang1234.sm@outlook.com (T.W.); gywang@cmu.edu.cn (G.W.); fhzhao@cmu.edu.cn (F.Z.); 2 Department of Environmental and Occupational Health, School of Public Health, Shenyang Medical University, Shenyang 110122, China; jinxxsmc@outlook.com

**Keywords:** 1,2-Dichloroethane poisoning, reactive astrocytes, microglial polarization, neuroinflammation, proinflammatory factors, brain edema formation

## Abstract

We have previously reported that the activation of astrocytes and microglia may lead to the overproduction of proinflammatory mediators, which could induce neuroinflammation and cause brain edema in 1,2-dichloroethane (1,2-DCE)-intoxicated mice. In this research, we further hypothesized that astrocyte–microglia crosstalk might trigger neuroinflammation and contribute to brain edema in 1,2-DCE-intoxicated mice. The present research revealed, for the first time, that subacute intoxication with 1,2-DCE might provoke the proinflammatory polarization of microglia, and pretreatment with minocycline, a specific inhibitor of microglial activation, may attenuate the enhanced protein levels of ionized calcium-binding adapter molecule1 (Iba-1), cluster of differentiation 11b (CD11b), glial fibrillary acidic protein (GFAP), soluble calcium-binding protein 100B (S100B), tumor necrosis factor α (TNF-α), interleukin 6 (IL-6), inducible nitric oxide synthase (iNOS), vascular cell adhesion molecule-1 (VCAM-1), intercellular adhesion molecule-1 (ICAM-1), matrix metalloproteinase-9 (MMP-9), Toll-like receptor 4 (TLR4), MyD88, and p-p65, and ameliorate the suppressed protein expression levels of occludin and claudin 5; we also observed changes in water content and made pathological observations on edema in the brains of 1,2-DCE-intoxicated mice. Moreover, pretreatment with fluorocitrate, an inhibitor of reactive astrocytes, could also reverse the alteration in protein expression levels of GFAP, S100B, Iba-1, CD11b, TNF-α, IL-6, iNOS, VCAM-1, ICAM-1, MMP-9, occludin, and claudin 5 in the brain of 1,2-DCE intoxicated mice. Furthermore, pretreatment with melatonin, a well-known anti-inflammatory drug, could also attenuate the above-mentioned changes in the brains of 1,2-DCE-intoxicated mice. Altogether, the findings from this research indicated that microglial activation might play an important role in triggering neuroinflammation, and hence may contribute to brain edema formation; additionally, the findings suggested that molecular crosstalk between reactive astrocytes and activated microglia may amplify the neuroinflammatory reaction, which could induce secondary brain injury in 1,2-DCE-intoxicated mice.

## 1. Introduction

1,2-Dichloroethane (1,2-DCE), a synthetic halogenated hydrocarbon, is applied to the manufacture of polyvinyl chloride in the plastics industry, but it can cause brain edema under subacute exposure [1,2]. We previously found that neuroinflammation might be involved in matrix metalloproteinase-9 (MMP-9) upregulation, blood–brain barrier (BBB) damage, and edema formation in the brains of 1,2-DCE-intoxicated mice [3]. Studies up to now have demonstrated that neuroinflammation is associated with the pathogenesis of many brain diseases, and that it compounds neurotoxicity [4]. Emerging evidence indicates that crosstalk between microglia and astrocytes is fundamental to triggering neuroinflammation, and determines the fate of brain injury [5,6]. By releasing different signaling molecules, both microglia and astrocytes establish autocrine feedback and their bidirectional conversation for a tight reciprocal modulation during brain injury [7]. Thus, microglia–astrocyte crosstalk is important for regulating microglial phenotypes and astrocytic functions, and is the determinant of the degree and duration of neuroinflammatory responses [8].

Microglia, as primary innate immune cells, play crucial roles in the response to injury within the brain [9]. Any disturbances in the brain microenvironmental homeostasis immediately lead to their activation, proliferation, and morphological alteration [10,11]. Microglial activation is frequently observed in a variety of neurological diseases, including neurodegeneration, neurotoxicity, and cerebral injury. As a myeloid-derived cell, microglia can polarize into the two kinds of phenotypes upon activation [12,13]. The proinflammatory phenotype promotes the inflammatory responses by releasing proinflammatory mediators [14]. Many studies have revealed that astrocytes are activated after microglial polarization [15]. However, astrocytes can be stimulated under some pathological conditions and release a series of proinflammatory mediators [16]. Along with advances in the conceptual and technological understanding of their biology, astrocytes are increasingly viewed as having a critical contribution to neurological diseases [17].

As the most abundant cells in the brain, astrocytes play an indispensable role in the survival and function of neurons by maintaining BBB integrity and extracellular environmental homeostasis [18]. Since astrocytes directly adhere to the endothelial cells of cerebral capillaries, they are an indispensable component of the BBB [19]. Due to high lipid solubility, 1, 2-DCE in the peripheral circulation can easily pass through the BBB, and thus astrocytes may be the first target of, as well as early respondents to, 1,2-DCE [20]. On the other hand, astrocytes are an important provider of several proinflammatory mediators [21]. Therefore, it is essential to understand the changes in the polarization of microglia following astrocyte activation. Thus far, the essential molecular crosstalk between reactive astrocytes and activated microglia is unclear in 1,2-DCE-induced brain edema. As far as we know, this is the first piece of research to explore the role of crosstalk between astrocytes and microglia in brain edema caused by 1,2-DCE poisoning in mice.

## 2. Materials and Methods

### 2.1. Animals

Female mice (Kunming species, albino) aged 3–4 weeks were obtained from the Animal Management Laboratory of China Medical University. The temperature and humidity in the animal room were 22–24 °C and 50%–60%. The animals were weighed and their poisoning status was observed every day during the experiment. The present experimental procedures were approved by the Committee for Animal Protection and Utilization of China Medical University, which conformed to the requirements of the China National Laboratory Animal Protection Guidelines. The experiment identification number is IACUC: 201910009.

### 2.2. Reagents

The 1,2-dichloroethane was purchased from Sinopharm Chemical Reagent Co., Ltd. (Ningbo, China). The BCA protein assay kit, multicolor prestained protein marker, and ECL plus kit were obtained from Thermo Fisher Scientific (Waltham, MA, USA). Antibodies for claudin 5 and occludin were obtained from Abcam (Cambridge, UK). Antibodies against glial fibrillary acidic protein (GFAP) and soluble calcium-binding protein 100B (S100B) were the products of ABMART (Shanghai, China). Antibodies against ionized calcium-binding adapter molecule1 (Iba-1), interleukin 6 (IL-6), cluster of differentiation 11b (CD11b), and nuclear factor-κB (NF-κB, p-p65) were obtained from ImmunoWay (Texas, USA). Antibodies for tumor necrosis factor α (TNF-α) and β-actin were purchased from Elabscience (Wuhan, China). Antibodies for arginase-1 (Arg-1), intercellular adhesion molecule-1 (ICAM-1), vascular cell adhesion molecule-1 (VCAM-1), MMP-9, inducible nitric oxide synthase (iNOS), Toll-like receptor 4 (TLR4), and MyD88 were obtained from Wanleibio (Shenyang, China). RIPA lysis buffer and the PAGE Gel Kit were obtained from EpiZyme (Shanghai, China). Minocycline was purchased from Solarbio (Beijing, China). DL-Fluorocitric acid barium salt and melatonin were obtained from Sigma-Aldrich (St. Louis, MO, USA). 

### 2.3. Experimental Procedures and Treatment

The experiment performed in the present study included four sections, which were designed to investigate the effects of 1,2-DCE on microglial polarization and the molecular crosstalk between microglia and astrocytes when neuroinflammation was triggered in 1,2-DCE-intoxicated mice. Static inhalation exposure was adopted in the experiment, and five mice were placed in every chamber with a volume of 100 L. 1,2-DCE was added to a plate suspended in the chamber and evaporated quickly by a fan after sealing. During exposure, contents of carbon dioxide, oxygen, and 1,2-DCE in the chamber were detected every hour. The merits and shortcomings of static inhalation exposure have been described in our previous papers. At the end of exposure, the concentrations of carbon dioxide, oxygen, and humidity in the chamber were lower than 1.5%, close to 20% and less than 70%, respectively. In addition, the equivalent concentration of 1, 2-DCE in the chamber during exposure was close 1.00 mg/L.

Mice were first given a week of adaptive feeding, and then those in the poisoned and intervention groups were exposed to 1.2 mg/L 1,2-DCE for 3.5 h per day up to three days. Mice in both the negative control and inhibitor control groups were treated the same without 1,2-DCE exposure. To investigate the effects of activated microglia on 1,2-DCE-induced brain edema, mice in the intervention group were intraperitoneally administrated with 45 mg/kg bodyweight (b.w) of minocycline in 200 µL normal saline, 1 h before every 1,2-DCE exposure.

In addition, to investigate the roles of reactive astrocytes in 1,2-DCE-induced neuroinflammation and BBB destruction, mice in the intervention group were pretreated with melatonin or fluorocitrate by intraperitoneal and intracerebroventricular injection, respectively. Melatonin was injected at a dosage of 20 mg/kg dissolved in 200 µL saline with 10% Tween-80 and 5% dimethyl sulfoxide (DMSO), 2 h before every 1,2-DCE exposure. Fluorocitrate was given at a dosage of 1 nmol/mouse in 5 μL saline, 2 h ahead of 1,2-DCE exposure. Mice in the control and intoxicated groups were pretreated with the corresponding solvent, and the inhibitor control group were pretreated with the inhibitor. 

For intracerebroventricular injection, mice were deeply anesthetized by 1% pentobarbital sodium (100 mg/kg), and then a tiny borehole in the dextral skull using the stereotaxic apparatus was perforated. The stainless steel guide cannula was implanted into the right lateral ventricle (1.2 mm horizontal to bregma, 2.5 mm below the skull, and 0.8 mm posterior). Fluorocitrate was injected at a rate of 1.0 μL/min with a microsyringe, and the needle was left for 10 min to permit diffusion of the liquor after injection.

Mice in the first part of the experiment were sacrificed at 24 h after one-, two-, and three-day exposure. In parts two to four of the experiment, they were sacrificed 24 h after the three-day exposure (anesthetized by 1% pentobarbital sodium 100 mg/kg b.w). Ten mice were in each group, and their brains were removed promptly to a cold plate and kept in a −80 °C refrigerator.

### 2.4. Analysis 

#### 2.4.1. Brain Water Content

In brief, the brain tissues were dissected immediately and weighed with a chemical balance, which was recorded as the wet weight. Next, the specimens were dried in an oven at 10 °C for 48 h to acquire the dry weight. The brain water content was computed by the following equation: [(wet weight – dry weight)/wet weight] × 100%.

#### 2.4.2. Histological Observation

After cardiac perfusion, the brains of the mice were fixed using 4% paraformaldehyde overnight and embedded in paraffin. Coronal sections of 5 μm were sliced and stained with hematoxylin and eosin (H&E staining). Reagents were obtained from Beyotime Biotechnology (Shanghai, China).

#### 2.4.3. Western Blot

Cerebral cortices were homogenized and lysed in the RIPA buffer, and the protein contents in the lysates were determined by the BCA protein assay kit. An equal number of proteins were separated on SDS-PAGE, and then transferred to PVDF membranes (Millipore, Bedford, MA, USA). The membranes were immersed with 5% skim milk and probed with primary antibodies of rabbit anti-mouse against Iba-1 (1:1000, YN2165), CD11b (1:1000, YT5660), Arg-1 (1:1000, WL02825), GFAP (1:1000, T55424), S100B (1:1000, T55201), MMP-9 (1:1000, WL03096), TNF-α (1:1000, E-AB-40015), IL-6 (1:1000, DF6087), iNOS (1:1000, WL0992a), ICAM-1 (1:1000, WL02268), VCAM-1 (1:1000, WL02474), NF-κB (p-p65) (1:1000, YP0191), TLR4 (1:1000, WL00196), MyD88 (1:1000, WL02494), occludin (1:1000, ab167161), claudin 5 (1:1000, ab131259), and β-actin (1:1000, E-AB-40338) at 4 °C overnight. The next morning, membranes were hatched with the secondary antibody of goat anti-rabbit at 4 °C for 1 h. The protein signals were detected with the ECL plus kit and photographed using Azure c500. Finally, proteins were quantified using ImageJ software 1.8.0 (Bio-Rad, Hercules, CA, USA) and expressed as the relative levels normalized to β-actin.

#### 2.4.4. ELISA

The lysates of cerebral tissues were centrifuged at 12,000 rpm for 10 min, and then the contents of TNF-α and IL-6 in the supernatant were measured using the specific ELISA kits based on the manufacturer’s instructions. TNF-α and IL-6 ELISA kits were obtained from Elabscience (Wuhan, China). 

### 2.5. Statistical Analysis

All data were presented as means ± standard deviations (SD) and were statistically analyzed using SPSS 22.0. Statistical comparisons of data among groups of different exposure days were carried out by one-way analysis of variance (ANOVA) followed by the Student–Newman–Keuls (SNK) test. Student’s unpaired *t*-tests were used to evaluate the difference between the 1,2-DCE-intoxicated groups with and without the preventive agents. A *p*-value under 0.05 was accepted as statistically significant.

## 3. Results

### 3.1. Effects of 1,2-DCE on Microglial Polarization during the Process of Brain Edema Formation in Mice 

**Figure 1:**
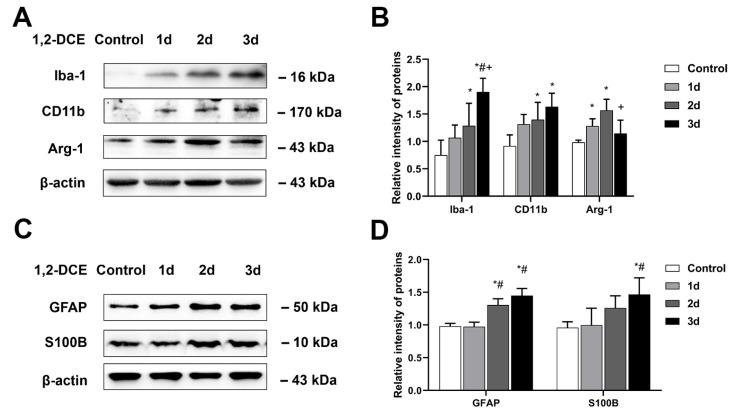
Effects of subacute poisoning with 1,2-DCE on the activation of microglia and astrocytes in the brains of mice. (**A**,**B**) Representative bands of Iba-1, CD11b, and Arg-1, as well as their quantification by Western blotting analysis. (**C**,**D**) Representative bands of GFAP, and S100B, as well as their quantification by Western blotting analysis. Notes: The square plots show the relative levels of target proteins among different groups, which were standardized by β-actin. *n* = 5, mean ± SD, one-way ANOVA followed by SNK tests. *p* < 0.05, *, vs. control group; #, vs. one-day exposure group; +, vs. two- day exposure group. Iba-1, ionized calcium-binding adapter molecule 1; CD11b, cluster of differentiation 11b; Arg-1, arginase-1; GFAP, glial fibrillary acid protein; S100B, soluble calcium-binding protein 100B.

In this part of the experiment, the control and the one-, two- and three-day exposure groups were divided. Mice were exposed to 0 and 1.2 mg/L 1,2-DCE for one, two, and three days, respectively. The protein expression levels of Iba-1, and CD11b in the mouse brains of the two- and three-day exposure groups significantly increased by contrast with the control group, and those of Iba-1 in the three-day exposure group were significantly higher than in the other exposure groups. While the protein levels of Arg-1 in the mouse brains of the one- and two-day exposure groups were significantly increased compared to the control, those in the three-day exposure group were significantly reduced compared to the two-day exposure groups, and did not differ significantly with the control group (Figure 1A,B). Moreover, the protein expression levels of GFAP and S100B in the mouse brains of the three-day exposure group increased significantly compared with the control and the one-day exposure group, and those of GFAP in the two-day exposure group were also significantly increased compared to the control and the one-day exposure group (Figure 1C,D). These results revealed that subacute poisoning with 1,2-DCE could activate both astrocytes and microglia, and finally stimulate the proinflammatory polarization of microglia in mice.

**Figure 2:**
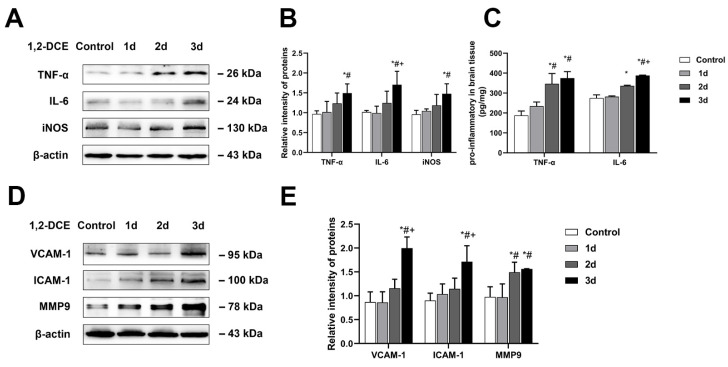
Effects of subacute poisoning with 1,2-DCE on the protein expression of proinflammatory mediators in the brains of mice. (**A**,**B**) Representative bands of TNF-α, IL-6, and iNOS, as well as their quantification by Western blotting analysis. (**C**) Protein levels of matured forms of TNF-α and IL-6 in the brain homogenate measured by ELISA kits. (**D**,**E**) Representative bands of VCAM-1, ICAM-1, and MMP-9, as well as their quantification by Western blotting analysis. Notes: The square plots show the relative levels of target proteins among different groups, which were standardized by β-actin. *n* = 5, mean ± SD, one-way ANOVA followed by SNK tests. *p* < 0.05, *, vs. control group; #, vs. one-day exposure group; +, vs. two-day exposure group. TNF-α, tumor necrosis factor alpha; IL-6, interleukin-6; iNOS, inducible nitric oxide synthase; VCAM-1, vascular cell adhesion molecule-1; ICAM-1, intercellular adhesion molecule-1; MMP-9, matrix metalloproteinase-9.

In addition, the protein expression levels of TNF-α, IL-6, and iNOS in the mouse brains of the three-day exposure group were significantly upregulated compared to the control and the one-day exposure groups, and those of IL-6 were significantly higher than in the two-day exposure group (Figure 2A,B). Similarly, the levels of the mature form of TNF-α and IL-6 in the brain homogenate of the two- and three-day exposure- groups significantly increased compared with the control group, and those of TNF-α were also significantly higher than in the one-day exposure group. Those of IL-6 in the three-day exposure group were significantly higher than in the other exposure groups (Figure 2C). Furthermore, the protein expression levels of ICAM-1, VCAM-1 and MMP-9 in the mouse brains of the three-day exposure group were significantly enhanced compared to the control and the one-day exposure groups, and those of ICAM-1 and VCAM-1 were also significantly higher than in the two-day exposure group. Those of MMP-9 in the two-day exposure group were also significantly enhanced compared to the control and the one-day exposure groups (Figure 2D,E). These data indicate that subacute poisoning with 1,2-DCE could trigger the neuroinflammation in mice.

### 3.2. Roles of Activated Microglia in 1,2-DCE-Induced Neuroinflammation and Brain Edema in Mice

In order to investigate the roles of activated microglia in 1,2-DCE-induced cerebral edema, mice were pretreated with minocycline, a widely used inhibitor of microglial activation, before every 1,2-DCE exposure. In this part of the experiment, we divided them into four groups: control, inhibitor, poisoning, and intervention. Mice were pretreated with saline in the control and poisoning groups, and minocycline in the inhibitor control and intervention groups, respectively. Then, the mice in the poisoning and intervention groups were exposed to 1,2-DCE for three days as mentioned above. 

**Figure 3:**
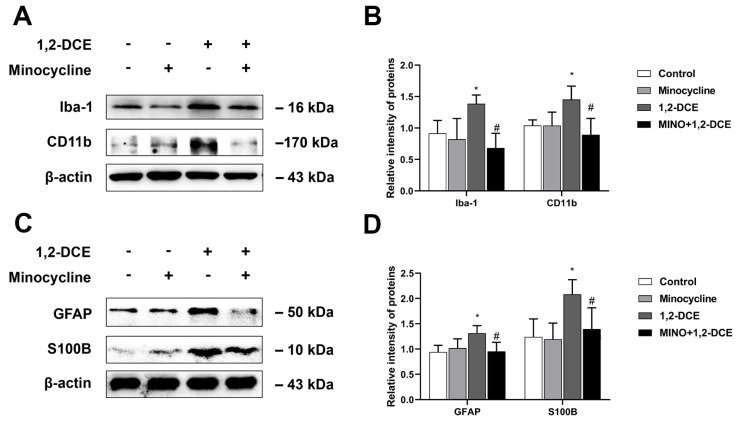
Pretreatment with minocycline on activation of microglia and astrocytes in the brains of 1,2-DCE-intoxicated mice. (**A**,**B**) Representative bands of Iba-1, and CD11b, as well as their quantification by Western blotting analysis. (**C**,**D**) Representative bands of GFAP, and S100B, as well as their quantification by Western blotting analysis. Notes: The square plots show the relative levels of target proteins among different groups, which were standardized by β-actin. *n* = 5, mean ± SD, Student’s unpaired *t*-test. *p* < 0.05, *, between 1,2-DCE intoxicated group and control group; #, between 1,2-DCE intoxicated group and intervention group. Iba-1, ionized calcium-binding adapter molecule 1; CD11b, cluster of differentiation 11b; GFAP, glial fibrillary acid protein; S100B, soluble calcium-binding protein 100B.

**Figure 4:**
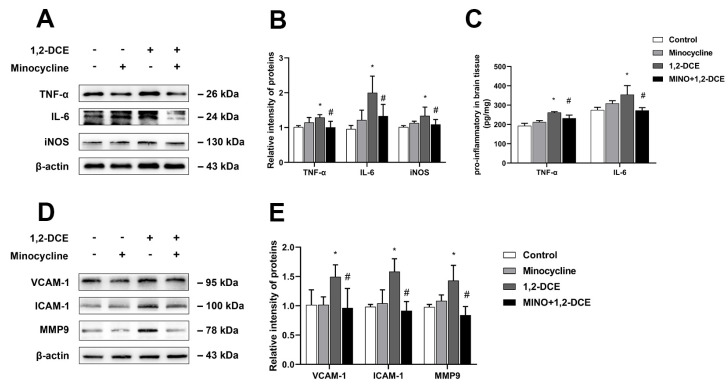
Pretreatment with minocycline on protein expression of proinflammatory mediators in the brains of 1,2-DCE-intoxicated mice. (**A**,**B**) Representative bands of TNF-α, IL-6, and iNOS, as well as their quantification by Western blotting analysis. (**C**) Protein levels of matured forms of TNF-α and IL-6 in the brain homogenate measured by ELISA kits. (**D**,**E**) Representative bands of VCAM-1, ICAM-1, and MMP-9, as well as their quantification by Western blotting analysis. Notes: The square plots show the relative levels of target proteins among different groups, which were standardized by β-actin. *n* = 5, mean ± SD, Student’s unpaired *t*-test. *p* < 0.05, *, between 1,2-DCE intoxicated group and control group; #, between 1,2-DCE intoxicated group and intervention group. TNF-α, tumor necrosis factor alpha; IL-6, interleukin-6; iNOS, inducible nitric oxide synthase; VCAM-1, vascular cell adhesion molecule-1; ICAM-1, intercellular adhesion molecule-1; MMP-9, matrix metalloproteinase-9.

**Figure 5:**
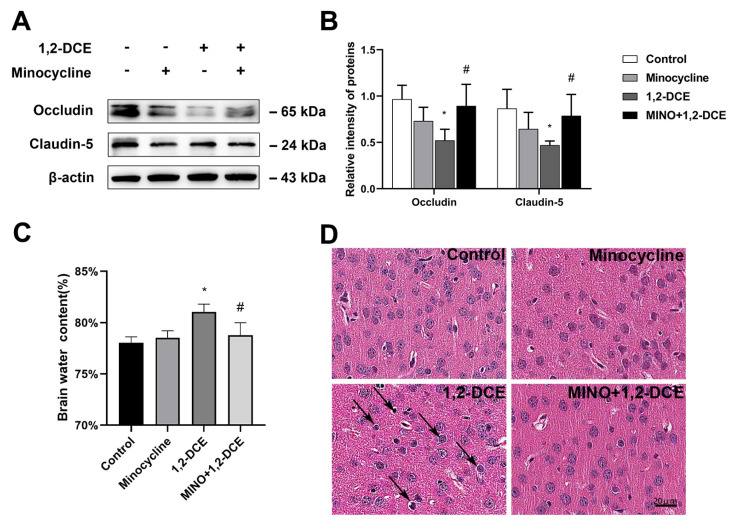
Pretreatment with minocycline on blood–brain barrier disruption and brain edema formation in 1,2-DCE-intoxicated mice. (**A**,**B**) Representative bands of occludin and claudin 5, as well as their quantification by Western blotting analysis. (**C**) Comparison of brain water contents in mice among different groups. (**D**) The typical images of pathological observation in the frontoparietal region of cerebral cortex stained by HE (200×); the scale bar represents 20 μm. Notes: The square plot shows the relative levels of target proteins among different groups, which were standardized by β-actin. *n* = 5, mean ± SD, Student’s unpaired *t*-test. *p* < 0.05, *, between 1,2-DCE intoxicated group and control group; #, between 1,2-DCE intoxicated group and intervention group. Arrows indicated enlarged perinuclear spaces and widened lacunar surrounding vessels.

Pretreatment with minocycline significantly reversed the alterations in the protein levels of Iba-1, CD11b, GFAP, S100B, TNF-α, IL-6, iNOS, VCAM-1, ICAM-1, and MMP-9 in the brain of 1,2-DCE-intoxicated mice (Figure 3A–D and Figure 4A–E). Moreover, minocycline pretreatment could also ameliorate the alterations in protein levels of claudin 5 and occludin, as well as water contents, and the pathological changes of edema in the brains of 1,2-DCE-intoxicated mice (Figure 5A–D). Altogether, the above results suggested that microglial activation might play a key role in neuroinflammation, BBB destruction, and cerebral edema formation in 1,2-DCE-intoxicated mice.

### 3.3. Role of TLR4/MyD88/NF-κB Signaling Pathway in Microglial Activation in 1,2-DCE-Intoxicated Mice

**Figure 6:**
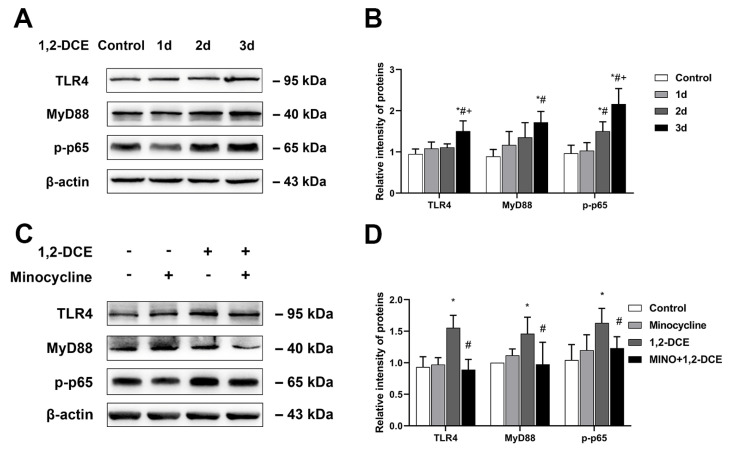
Role of TLR4/MyD88/NF-κB signaling pathway in microglial activation the brains of 1,2-DCE-intoxicated mice. (**A**,**B**) Representative bands of TLR4, MyD88, and p-p65, as well as their quantification by Western blotting analysis among different exposure groups. (**C**,**D**) Representative bands of TLR4, MyD88, and p-p65, as well as their quantification by Western blotting analysis among different pretreated groups. Notes: The square plots show the relative levels of target proteins among different groups, which were standardized by β-actin. *n* = 5, mean ± SD, one-way ANOVA followed by SNK tests. *p* < 0.05, *, vs. control group; #, vs. one-day exposure group; +, vs. two-day exposure group, in the upper panel, and Student’s unpaired *t*-test. *p* < 0.05, *, between 1,2-DCE intoxicated group and control group; #, between 1,2-DCE intoxicated group and intervention group, in the lower panel.

As shown in Figure 6A,B, the protein levels of TLR4, MyD88, and p-p65 in the mouse brains of the three-day exposure group increased significantly compared to the control and one-day exposure groups, and those of TLR4 and p-p65 were also significantly higher than in the two-day exposure group. Those of p-p65 in the two-day exposure group significantly increased compared to the control and one-day exposure groups. Moreover, the minocycline pretreatment could significantly suppress the enhanced protein levels of TLR4, MyD88, and p-p65 in the brains of 1,2-DCE-intoxicated mice (Figure 6C,D), suggesting that TLR4/MyD88/NF-κB signaling pathway was probably involved in microglial activation.

### 3.4. Roles of Reactive Astrocytes in Microglial Activation and Neuroinflammation in 1,2-DCE-Intoxicated Mice 

**Figure 7:**
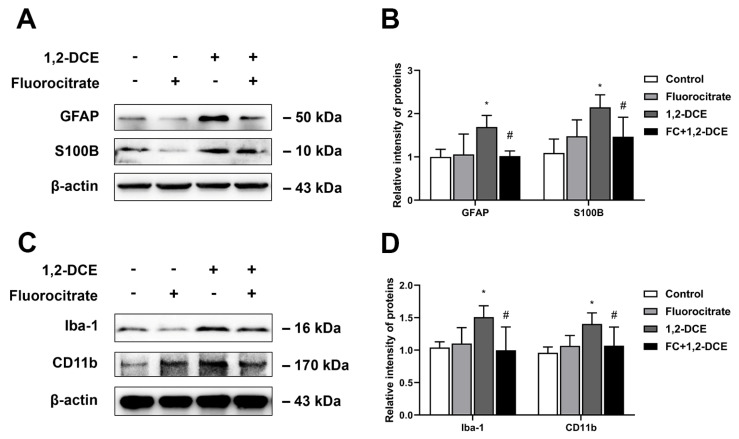
Pretreatment with fluorocitrate on activation of astrocytes and microglia in the brains of 1,2-DCE-intoxicated mice. (**A**,**B**) Representative bands of GFAP, and S100B, as well as their quantification by Western blotting analysis. (**C**,**D**) Representative bands of Iba-1, and CD11b, as well as their quantification by Western blotting analysis. Notes: The square plots show the relative levels of target proteins among different groups, which were standardized by β-actin. *n* = 5, mean ± SD, Student’s unpaired *t*-test. *p* < 0.05, *, between 1,2-DCE intoxicated group and control group; #, between 1,2-DCE intoxicated group and intervention group. Iba-1, ionized calcium-binding adapter molecule 1; CD11b, cluster of differentiation 11b; GFAP, glial fibrillary acid protein; S100B, soluble calcium-binding protein 100B.

**Figure 8:**
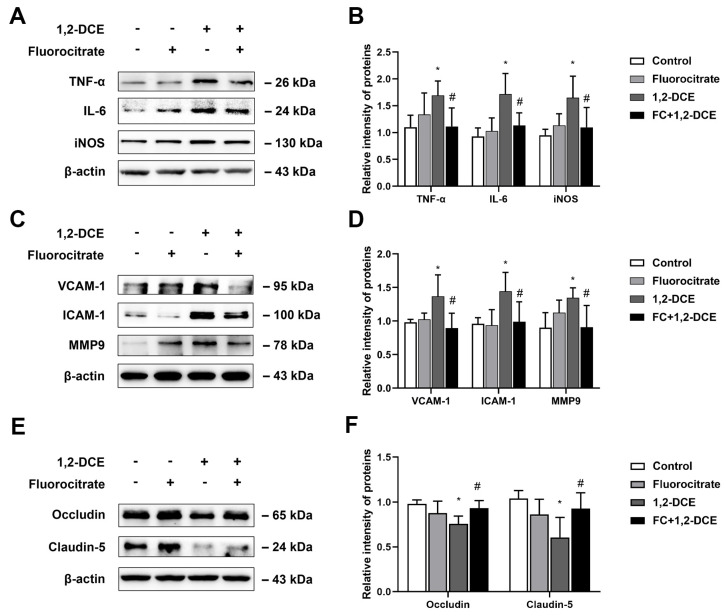
Pretreatment with fluorocitrate on protein expression of proinflammatory mediators, cell adhesion molecules, and tight junction proteins in the brains of 1,2-DCE-intoxicated mice. (**A**,**B**) Representative bands of TNF-α, IL-6, and iNOS, as well as their quantification by Western blotting analysis. (**C**,**D**) Representative bands of VCAM-1, ICAM-1, and MMP-9, as well as their quantification by Western blotting analysis. (**E**,**F**) Representative bands of occludin and claudin 5, as well as their quantification by Western blotting analysis. Notes: The square plots show the relative levels of target proteins among different groups, which were standardized by β-actin. *n* = 5, mean ± SD, Student’s unpaired *t*-test. *p* < 0.05, *, between 1,2-DCE intoxicated group and control group; #, between 1,2-DCE intoxicated group and intervention group. TNF-α, tumor necrosis factor alpha; IL-6, interleukin-6; iNOS, inducible nitric oxide synthase; VCAM-1, vascular cell adhesion molecule-1; ICAM-1, intercellular adhesion molecule-1; MMP-9, matrix metalloproteinase-9.

**Figure 9:**
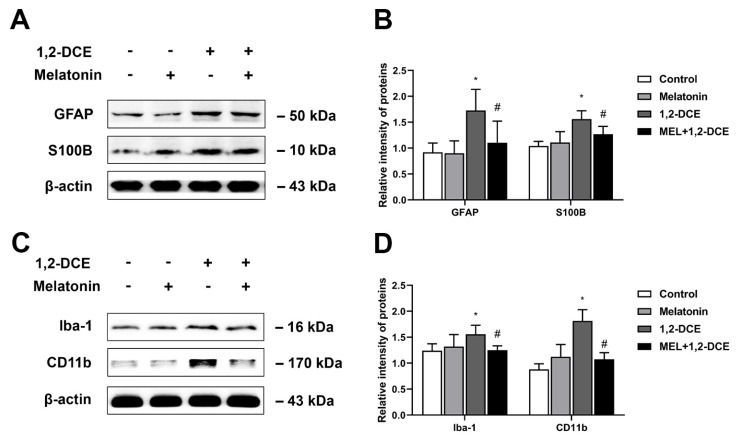
Pretreatment with melatonin on activation of astrocytes and microglia in the brains of 1,2-DCE-intoxicated mice. (**A**,**B**) Representative bands of GFAP and S100B, as well as their quantification by Western blotting analysis. (**C**,**D**) Representative bands of Iba-1, and CD11b, as well as their quantification by Western blotting analysis. Notes: The square plots show the relative levels of target proteins among different groups, which were standardized by β-actin. *n* = 5, mean ± SD, Student’s unpaired *t*-test. *p* < 0.05, *, between 1,2-DCE intoxicated group and control group; #, between 1,2-DCE intoxicated group and intervention group. Iba-1, ionized calcium-binding adapter molecule 1; CD11b, cluster of differentiation 11b; GFAP, glial fibrillary acid protein; S100B, soluble calcium-binding protein 100B.

**Figure 10:**
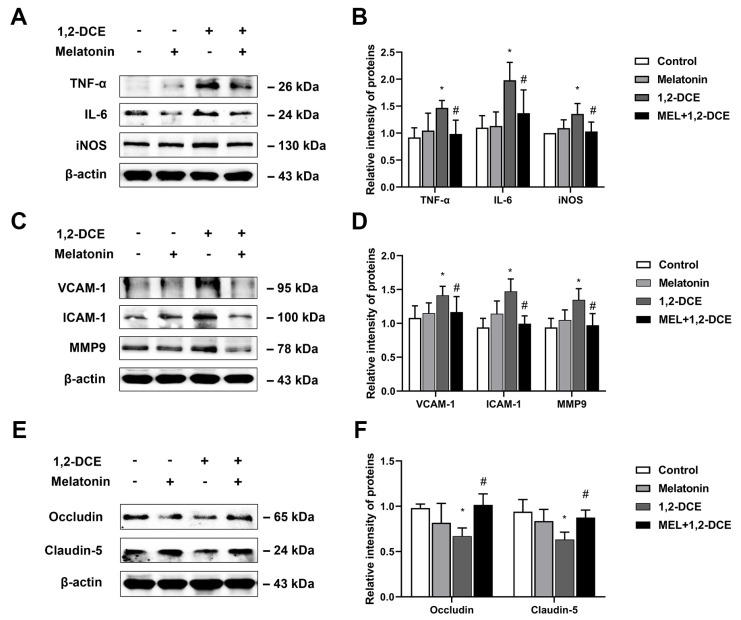
Pretreatment with melatonin on protein expression of proinflammatory mediators, cell adhesion molecules, and tight junction proteins in the brains of 1,2-DCE-intoxicated mice. (**A**,**B**) Representative bands of TNF-α, IL-6, and iNOS, as well as their quantification by Western blotting analysis. (**C**,**D**) Representative bands of VCAM-1, ICAM-1, and MMP-9, as well as their quantification by Western blotting analysis. (**E**,**F**) Representative bands of occludin and claudin 5, as well as their quantification by Western blotting analysis. Notes: The square plots show the relative levels of target proteins among different groups, which were standardized to β-actin. *n* = 5, mean ± SD, Student’s unpaired *t*-test. *p* < 0.05, *, between 1,2-DCE intoxicated group and control group; #, between 1,2-DCE intoxicated group and intervention group. TNF-α, tumor necrosis factor alpha; IL-6, interleukin-6; iNOS, inducible nitric oxide synthase; VCAM-1, vascular cell adhesion molecule-1; ICAM-1, intercellular adhesion molecule-1; MMP-9, matrix metalloproteinase-9.

To investigate the crosstalk between astrocytes and microglia, and the effects of reactive astrocytes on 1,2-DCE-induced neuroinflammation and BBB destruction, mice were pretreated with fluorocitrate or melatonin before 1,2-DCE exposure. Pretreatment with either fluorocitrate or melatonin could markedly reverse the alterations in protein expression levels of GFAP, S100B, Iba-1, CD11b, TNF-α, IL-6, iNOS, ICAM-1, VCAM-1, MMP-9, occludin, and claudin 5 in the brains of 1,2-DCE-intoxicated mice (Figure 7A–D, Figure 8A–F, Figure 9A–D and Figure 10A–F). Altogether, these data suggested that pretreatment with either fluorocitrate or melatonin could effectively suppress the reactive astrocytes and then abolish 1,2-DCE-induced microglial activation, neuroinflammation, and BBB destruction in the brains of mice. 

## 4. Discussion

We reported previously that brain edema could be induced in mice exposed to 1.2 mg/L 1,2-DCE for 3.5 h per day for up to three days [1,22]. During the course of brain edema formation, both microglia and astrocytes were activated, and the proinflammatory mediators, including IL-1β, MMP-9, iNOS, ICAM-1, and VCAM-1, were overproduced via activation of the p38 MAPK and NF-κB signaling pathway, which may trigger neuroinflammation and finally lead to BBB destruction in the brains of 1,2-DCE-intoxicated mice [16,23,24]. In the current study, we found for the first time that microglia could be polarized into the proinflammatory phenotypes during 1,2-DCE-induced brain edema, and microglial activation might be crucial for brain edema formation.

Neuroinflammatory reactions in response to intoxication, infection and trauma involve all the cell types in the brain, including neurons, microglia, and astrocytes, which may activate the glial cells, promote the release of proinflammatory mediators, destroy BBB integrity, and recruit peripheral immune cells [25,26,27,28,29]. Emerging evidence demonstrated that secondary degeneration to both damaged and healthy cells could be caused by microglia-mediated neuroinflammation [30]. Thus, microglia-mediated neuroinflammation is crucial for the process of brain injury and the final extent of impairment [31].

Iba-1, CD11b, and Arg-1 are widely used classical marker proteins specific to microglia in the brain [32]. Iba-1 is constitutively expressed by all resting and activated microglia, CD11b is the marker of proinflammatory polarization, and Arg-1 is the distinctive marker of neuroprotective polarization [33,34]. When activated, microglia can upregulate the gene expression of various proinflammatory factors and enhance the production of proinflammatory cytokines [35,36], which consequently stimulate the release of various inflammatory mediators by both astrocytes and microglia to induce neurotoxicity [37,38]. IL-6 and TNF-α, as well as nitric oxide (NO) produced by upregulated iNOS expression, are the earliest and most abundant proinflammatory factors released by activated microglia [39,40]. It is recognized that iNOS is not normally expressed in the brain but produces a dangerous amount of NO in the brain when induced in several pathological conditions [41]. Excessive release of these proinflammatory mediators is indicative of a clearly proinflammatory state [42].

TLR4, as a prime member of the pattern-recognition receptors, plays a crucial role in neuroinflammatory responses, which recognizes the products derived from damaged tissues termed damage-associated molecular patterns (DAMPs) [43]. After brain injury, sterile neuroinflammation is mainly triggered by DAMPs through TLR4/MyD88/NF-κB signaling in microglia [44]. Both VCAM-1 and ICAM-1 are the cell-adhesion molecules that belong to the immunoglobulin superfamily. They usually present at low levels on the luminal surface of endothelial cells in BBB. Under inflammatory conditions, the expression levels of VCAM-1 and ICAM-1 are upregulated and play a key role in the recruitment of peripheral immune cells [45]. MMP-9 belongs to the extracellular protease family, which is normally expressed at low levels, but overexpressed in many neurological diseases. In the brain, excessive MMP-9 can act not only as a proteolytic enzyme involved in BBB disruption, but also as a proinflammatory factor involved in the development of neuroinflammation, since BBB disruption allows the infiltration of peripheral immune cells into the brain parenchyma [3,46]. In addition, the tight junction proteins, commonly composed of occludin and claudin 5, are known to be indispensable components essential for the integrity of the BBB [47]. In the brain, abnormal expression of these proteins is indicative of inflammation and BBB integrity destruction.

The current study, together with our previous studies, indicated that the protein expression levels of Iba-1, CD11b, IL-6, TNF-α, iNOS, GFAP, S100B, VCAM-1, ICAM-1, and MMP-9 increased significantly, whereas those of the claudin 5 and occludin decreased markedly in the brains of 1,2-DCE-intoxicated mice [3], and pretreatment with the specific inhibitor of microglial activation could attenuate these changes [48,49]. Thus, our findings suggest that neuroinflammation was induced during brain edema, and microglial activation played a key role in triggering neuroinflammation. Additionally, the protein levels of TLR4, MyD88, and p-p65 were upregulated by 1,2-DCE, and minocycline reversed these changes in the brains of 1,2-DCE-intoxicated mice, indicating that the TLR4/MyD88/NF-κB signaling pathway could participate in microglial activation. Furthermore, inhibition of microglial activation could also reduce the water content and ameliorate the pathological changes of brain edema, indicating that microglia-mediated neuroinflammation leads to BBB destruction and brain edema. 

NF-κB is a transcription factor that can be activated by multiple signaling pathways, and then mediates inflammatory reactions by producing diverse proinflammatory cytokines, chemokines, and inducible enzymes [50]. In general, NF-κB is composed of p50 and p65 and is held in the cytoplasm in a non-activated state by combining with the inhibitory protein (IκB). The protein of p65 can be phosphorylated and translocated from the cytoplasm to the nucleus when IκB is degraded by the proteasome after phosphorylation [51]. Our previous studies found that the p38 MAPK/NF-κB signaling pathway could be activated, and it participated in the overexpression of MMP-9, ICAM-1, and VCAM-1, as well as BBB disruption and brain edema formation in 1,2-DCE-intoxicated mice [23]. In the current research, our findings demonstrated for the first time that subacute poisoning with 1,2-DCE could polarize microglia into the proinflammatory phenotype, and then microglial activation could promote astrocytic activation, and in turn trigger neuroinflammation and induce brain edema in 1,2-DCE-intoxicated mice.

**Figure 11:**
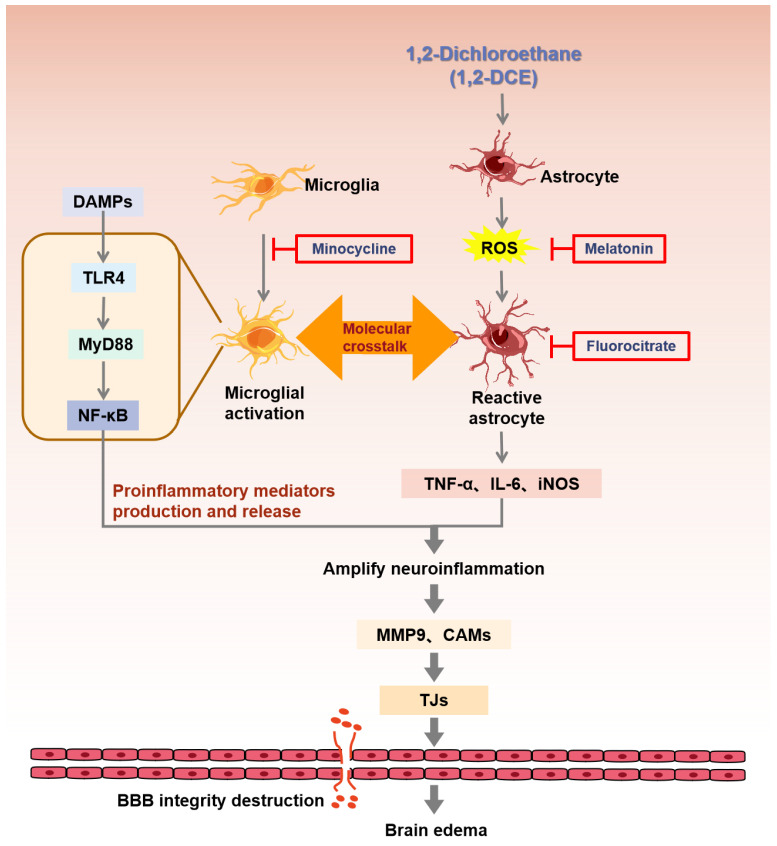
Schematic representation of the roles of microglia–astrocyte crosstalk in triggering neuroinflammation and brain edema in 1,2-DCE-intoxicated mice. 1,2-DCE with high lipid solubility can easily pass through BBB and activate astrocytes and microglia. Both activated microglia and reactive astrocytes can overproduce and release multiple proinflammatory mediators, such as TNF-α, IL-6, iNOS, MMP-9, and cell-adhesion molecules (CAMs). In microglia, the TLR4/MyD88/NF-κB signal pathway might be activated and may contribute to overproduction of proinflammatory mediators. The molecular crosstalk between microglia and astrocytes might amplify the neuroinflammatory reactions and, in turn, lead to secondary brain injury in 1,2-DCE-intoxicated mice. TNF-α, tumor necrosis factor alpha; IL-6, interleukin-6; iNOS, inducible nitric oxide synthase; MMP-9, matrix metalloproteinase-9; TLR4, Toll-like receptor 4; BBB, blood–brain barrier; TJs, tight junction proteins; DAMPs, damaged tissues termed damage-associated molecular patterns; ROS, Reactive Oxygen Species.

On the other hand, pretreatment with fluorocitrate or melatonin could also reverse the alterations in the protein expression levels of Iba-1, CD11b, GFAP, S100B, TNF-α, IL-6, and iNOS, as well as the cell-adhesion molecules and tight junction proteins in the brains of 1,2-DCE-intoxicated mice. It has been reported that fluorocitrate is preferentially taken up by astrocytes and can reversibly inhibit the tricarboxylic acid cycle by targeting aconitase [52]. Thus, it is thought to be a specific inhibitor of astrocytes. Melatonin is an anti-inflammatory drug with neuroprotective activity, which is most probably attributable to its biological functions in scavenging free radicals [53]. Treatment with melatonin could suppress the levels of IL-6, TNF-α, and IL-1β in animal models of brain ischemia/reperfusion injury, subarachnoid hemorrhage, and traumatic brain injury [54]. However, to date, there is no research to explore the inhibitory effects of fluorocitrate or melatonin on neuroinflammation associated with 1,2-DCE-induced brain edema. Therefore, our results demonstrated for the first time that the inhibition of reactive astrocytes could also suppress microglial activation and attenuate neuroinflammation in the brains of 1,2-DCE-intoxicated mice. The proposal schematic diagram was shown in Figure 11.

In conclusion, there were several novel findings from this study. First, we confirmed that subacute poisoning with 1,2-DCE in mice could stimulate the proinflammatory polarization of microglia. Second, the neuroinflammatory reaction in 1,2-DCE-intoxicated mice could be triggered either by microglial activation or reactive astrocytes. The most important findings from this study was that activation of microglia and astrocytes may lead to the overproduction of proinflammatory factors, which next activate more microglia and astrocytes and cause generation and release of more proinflammatory factors. The crosstalk between activated microglia and reactive astrocytes may amplify neuroinflammatory responses and in turn lead to secondary brain injury. Third, microglial activation could play a vital role in triggering neuroinflammation, and hence contribute to 1,2-DCE-induced brain edema formation. To conclude, the inhibition of neuroinflammatory reaction is expected to be a potential treatment to alleviate the progression of brain edema induced by subacute poisoning by 1,2-DCE. 

## Data Availability

All data are available from the corresponding author upon reasonable request.
